# Choropleth Mapping of Cervical Cancer Screening in South Africa Using Healthcare Facility-level Data from the National Laboratory Network

**DOI:** 10.3934/publichealth.2016.4.849

**Published:** 2016-10-19

**Authors:** Caroline B T Makura, Kathryn Schnippel, Pamela Michelow, Carla J. Chibwesha, Bridgette Goeieman, Suzette Jordaan, Cynthia Firnhaber

**Affiliations:** 1 Right to Care, Johannesburg, South Africa; 2 Clinical HIV Research Unit, Department of Clinical Medicine, Faculty of Health Sciences, University of the Witwatersrand, Johannesburg, South Africa; 3 Cytology Unit, Department of Anatomical Pathology, Faculty of Health Sciences, University of the Witwatersrand; 4 National Health Laboratory Service, Johannesburg, South Africa; 5 Department of Obstetrics and Gynecology, School of Medicine, University of North Carolina at Chapel Hill, Chapel Hill, North Carolina, USA

**Keywords:** cervical cancer screening, choropleth map, Pap smear, High grade squamous intraepithelial lesion (HSIL), South Africa

## Abstract

**Background:**

In South Africa, cervical cancer remains among the most common cancers and a leading cause of cancer death. Co-infection with HIV increases the risk of developing cervical pre-cancer and cancer. We analysed National Health Laboratory Service cervical cytology data to investigate geographic variations of Pap smear coverage, quality, and high grade lesions.

**Methods:**

Facility-level data were extracted from the NHLS for April 2013–March 2014. We present results and choropleth maps detailing coverage, adequacy and high-grade Pap smear cytology abnormalities defined as Pap smears suspicious for invasive carcinoma, high-grade squamous intraepithelial lesions (HSIL) or atypical squamous cells: cannot exclude HSIL (ASC-H).

**Results:**

4,562 facilities submitted 791,067 cytology slides. The interquartile range (IQR) for Pap smear coverage among HIV-infected women was 26–41%; similar to coverage in women aged 30 and older (IQR: 26–42%). 6/52 districts had adequacy rates above the national standard (70%) and 2/52 districts had adequacy rates below 35%. We observed marked variation in Pap smear abnormalities across the country, with the proportion of high-grade cytology abnormalities ≥0.3% in 17/52 districts.

**Conclusion:**

Using district-level choropleth maps, we are able to display variations in Pap smear coverage, quality, and results across South Africa. This approach may be used to improve resource allocation, achieving better equity in cervical cancer prevention.

## 1. Introduction

In South Africa, cervical cancer is the second most common cancer among women affecting 1 in every 41 and killing 8 women daily [1]; yet, it is one of the easily preventable cancers through vaccination, early detection and treatment of cervical cancer precursors [2]. Approximately 90% of cervical cancer deaths occurred in developing countries [3], highlighting inequality and existing barriers to access of cervical cancer population-based screening programs [4].

Since the introduction of the Papanicolaou (Pap) smear in the 1940s, it is estimated that there has been a 70% reduction in cervical cancer deaths in developed countries [5]. Conventional Pap smears are the standard of care for cervical cancer screening in South Africa's public healthcare system [6]. Current South African national health guidelines recommend for the general population, women over 30 years of age receive at least 3 Pap smears at 10-year intervals [4],[7]. The 2003 South Africa World Health Survey estimated the cervical cancer screening coverage to be under 20% for women aged 25 to 64 therefore a more organized cervical cancer is imperative [8].

HIV co-infection increases the risk of persistent infection with high-risk HPV (the necessary cause of most cervical cancers) and of cervical precancer and invasive cancer [4]. South Africa has approximately 3.5 million women living with HIV, one of the highest burdens of HIV infection in the world [9]. In response to the recognized risk of cervical disease among HIV-infected women, the South African Department of Health guidelines for HIV-infected women state that Pap smear screening should be done for all women at least 18 years of age at initiation of antiretroviral therapy (ART) and once every 3 years following a negative Pap result [10].

The South African National Health Laboratory Service (NHLS) is the country's largest diagnostic pathology service, serving the public health sector and approximately 80% of the total population. NHLS has 12 cytology laboratories located around South Africa in all 9 provinces. The NHLS central data warehouse manages data from all specimens submitted to the national laboratory network.

In this manuscript, we present district-level epidemiological analysis of the coverage, quality, and proportion of high-grade abnormal Pap smear results in South Africa using facility-level data on choropleth maps, which are maps that graphically represent different proportions of the measurement of interest.

## 2. Materials and Methods

This is a cross-sectional study and our primary objective was to create pictorial representations of Pap smear quality, coverage and high-grade abnormalities using NHLS cervical cytology data which is estimated to cover 80% of the population. For this study, NHLS provided routinely collected facility-level data for Pap smears for the period April 2013–March 2014.

Choropleth maps are thematic maps that can graphically represent different proportions of the measurement of interest by using differences in shading or colour to represent differences in results which were analysed. As such, we used choropleth maps to:

(1) Identify districts that were not reaching the national target of 70% for coverage of Pap smears screening; estimated for all women and HIV-infected women;

(2) Identify districts that were performing below the national standard of achieving a 70% Pap smear “adequacy” rate (defined as the proportion of specimens with endo-cervical cells noted as present among all specimens that were satisfactory for examination); and

(3) Identify districts with the highest proportion of Pap smear results suspicious for invasive carcinoma, with high-grade squamous intraepithelial lesions (HSIL) or atypical squamous cells: cannot exclude HSIL (ASC-H).

### 2.1. Choropleth Mapping

Choropleth maps were constructed in Stata version 14 (College Station, Texas) using the program spmap [11]. Boundary data for the 52 districts of South Africa was obtained as a shapefile from the Municipal Demarcation Board South Africa [12].

### 2.2. Pap Smear Coverage

During the period under study, South African cervical cancer screening guidelines recommended 3 Pap smears per lifetime for the general population: once every 10 years, beginning at age 30 [10]. South African HIV management guidelines for the study period indicated that HIV-infected women 18 years and older have a Pap smear screening at ART initiation and once every 3 years thereafter [10]. We aligned coverage benchmarks with those published by the South African Department of Health, for which the stated aim is to achieve Pap smear coverage rates of at least 70% [7].

Using the 2010 screening guidelines, we defined overall Pap smear coverage as proportion of Pap smears collected from women aged 30 years and older between April 2013 and March 2014 out of the number of eligible women aged 30 years and older and expected to screen in a 1-year period (i.e., one tenth of the female population aged 30 or older). The 2013 district mid-year population estimates from the census [13] were used to generate population denominators.

Similarly, Pap smear coverage in HIV-infected women was defined as the proportion of Pap smears estimated to have been collected from HIV-infected women out of the female population aged 25 and older estimated to be HIV-infected and expected to screen (i.e., one third of women estimated to be HIV-infected aged 25 years and older in each district correlating with the 2012 HIV provincial prevalence rates). HIV serostatus was available for 40% of the Pap smears recorded in the NHLS database; total Pap smears from HIV infected women were assumed to be those known to be HIV positive in addition to Pap smears approximated to be from HIV infected women by applying the HIV positivity rate (assumed to equal HIV infected women out of all women with an HIV status reported) to women whose HIV status was not known. For the denominator, the 2012 HIV provincial prevalence rates [14] for persons aged 25 years and older were applied to the female population to generate the estimated population of women aged 25 years or older living with HIV per district.

### 2.3. Pap Smear Quality

We present one measure of the quality of a Pap smear: smear “adequacy”, which is the adequacy rate defined as the proportion of specimens with endo-cervical cells noted as present among all specimens that were satisfactory for examination. Unsatisfactory specimens included those where the slide was broken, the label missing or unreadable, or the specimen was bloody or obscured. This portion of the analysis was not disaggregated by HIV serostatus. A local benchmark for Pap smear adequacy of least 70% in public healthcare facilities is stated in the South African cervical cancer screening guidelines [7] and was used in the analysis.

### 2.4. High-grade Cytological Abnormalities

We also mapped high-grade abnormalities reported. High-grade Pap smear cytology abnormalities were defined as Pap smears suspicious for invasive carcinoma, high-grade squamous intraepithelial lesions (HSIL), or atypical squamous cells: cannot exclude HSIL (ASC-H). Atypical glandular cells (AGC, formerly atypical glandular cells of undetermined significance or AGUS, were not included in the definition of high-grade cytology abnormalities although guidelines indicate colposcopic follow-up.

Very small number of Pap smears show AGC, the overwhelming majority of abnormalities are squamous (see Results) as AGC comprised 0.2% of abnormalities. The NHLS reports abnormalities using the Bethesda classification system [15]. The proportion of high-grade cytological abnormalities out of the overall total population estimate for women aged 25 and older were estimated for each district. Results were not disaggregated by HIV serostatus.

### 2.5. Ethics Statement

Data used for this study included facility-level counts reported from NHLS, as well as population and HIV prevalence estimates publically available from Statistics South Africa 2013 mid-year population estimates and South African 2012 National HIV Prevalence, Incidence and Behaviour Survey. No patient-level data were included. Database access to the NHLS cervical cancer data was granted by the NHLS Academics Affairs and Research office. A waiver confirming that the study did not include human subject research was received from the Human Research Ethics Committee of the Foundation for Professional Development in April 2016.

## 3. Results

Between April 2013 and March 2014 a total of 4,562 public-sector facilities from across all 52 South African districts submitted 791,067 cytology slides to the NHLS. Of those, 346,416 (44%) Pap smears were estimated to have been collected from HIV-infected woman and 420,319 (53%) were collected from women aged 30 years and older. The City of Johannesburg and Chris Hani districts (Gauteng and Eastern Cape respectively) had the most facilities reporting, with a respective share of 4.2% (n = 191) and 4.1% (n = 187) out of all the documented reporting facilities. The sparsely populated Central Karoo (Western Cape) and Amajuba districts (KwaZulu-Natal) had the fewest facilities out of all the facilities documented, with a reported share of 0.4% (n = 20) and 0.5% (n = 24), respectively.

### 3.1. Pap Smear Coverage

**Figure 1.**
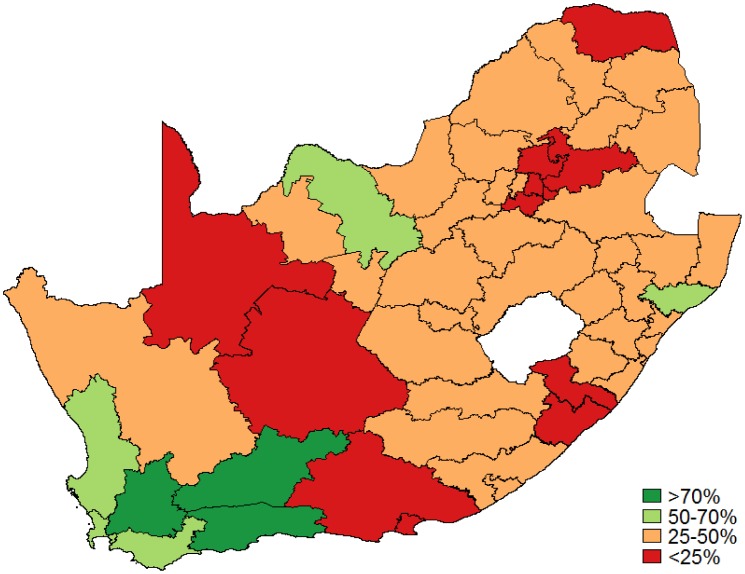
Pap smear coverage: In women aged 30 years and older.

**Figure 2.**
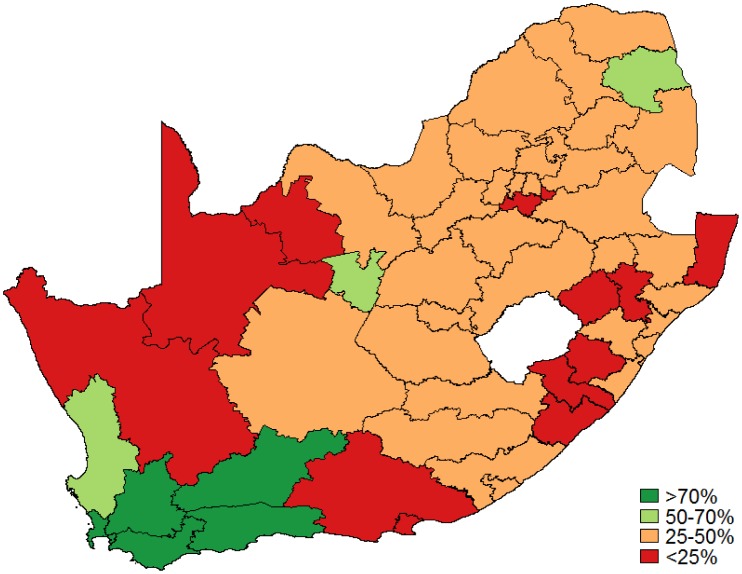
Pap smear coverage: HIV-infected women as a proportion of women aged 25 years and older.

Table 1 details the population estimate, total Pap smears collected, high-grade cytological results as a percentage of the total Pap smears collected and HIV-infection rate by district over one year. Figure 1 displays the Pap smear coverage rate in women aged 30 and older. The median coverage was 33% (IQR: 26–42%) with 44/52 districts below 50% coverage and 3/52 districts with at least 70% coverage. Figure 2 shows coverage in HIV-infected women as a proportion of women aged 25 and older estimated to be HIV infected with the median Pap smear coverage (once every 3 years) in HIV-infected women of 31% (IQR: 26–41%) with 44/52 districts below 50% coverage. Only 5 out of the 52 districts reached the target of 70% coverage for HIV-infected women.

### 3.2. Pap Smear Quality

**Figure 3.**
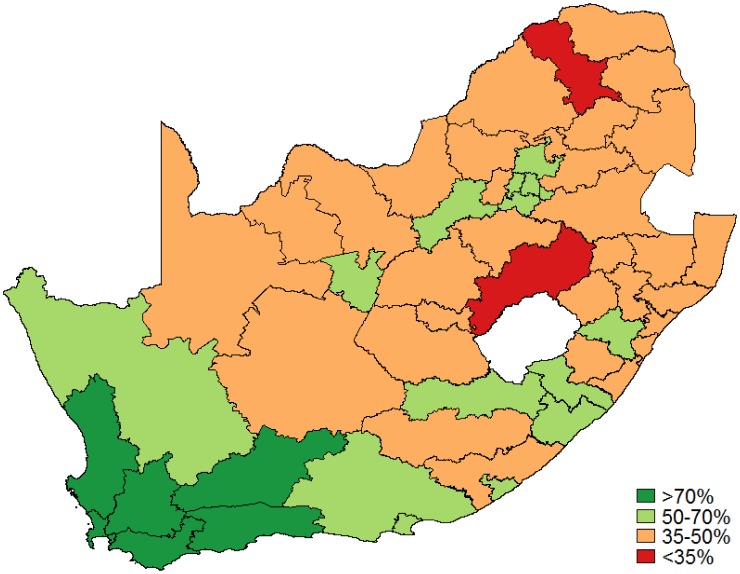
Pap smear adequacy: All women.

A total of 6 districts were above the national adequacy target of 70%, namely West Coast, Cape Winelands, Overberg, Central Karoo, Eden and City of Cape Town Metro (all from the Western Cape province) with adequacy rates between 71% and 79%. The median adequacy rate was 47% (IQR: 44–56%). Figure 3 also shows the varying adequacy rates across South Africa. Capricorn (Limpopo) and Thabo Mofutsanyana (Free State) districts reported the lowest rates with adequacy at 33% and 29%, respectively [10].

### 3.3. High-grade Cytological Abnormalities

**Figure 4.**
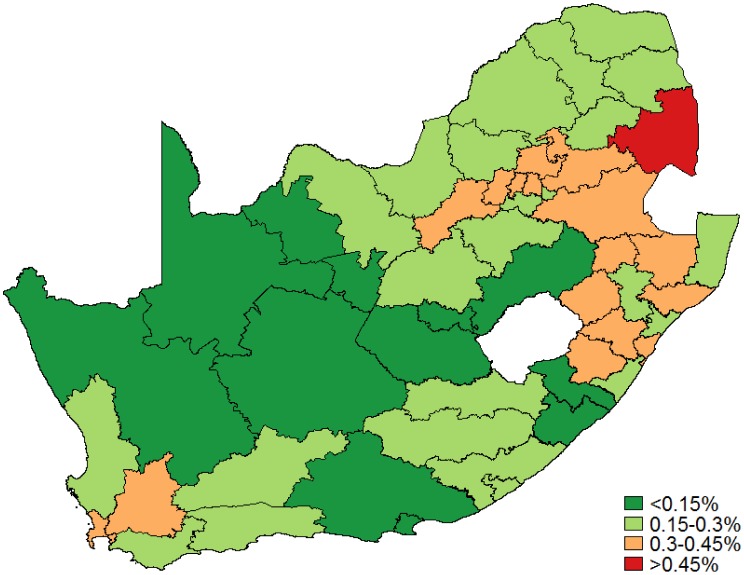
High-grade cytology abnormalities: All women as a proportion of women aged 25 years and older.

Overall, 43,346 (5.5%) of the Pap smear results were classified as high-grade abnormal cytology, including suspicious for invasive cervical cancer results. AGC results accounted for less than 0.2% of the total, and were not included in this classification. Figure 4 displays the proportions of high-grade results for each district as a proportion of women aged 25 and older. Cytology results are reported for all women and are not disaggregated by HIV serostatus. Once again, marked variation in Pap smear abnormalities is observed across the country, with the proportion of high-grade abnormalities ≥0.3% occurring in 17/52 districts in the general population of women.

**Table 1.** Population estimates, Pap smears done, HIV provincial rate and cytology results for all women, by district.

Province	District	Population estimate	Total No. of Pap smears	High-grade cytology abnormalities (%)	Provincial HIV %
**Eastern Cape**	Alfred Nzo	191,444	3,258	5.31	22
	Amathole	233,787	12,173	4.69	22
	Buffalo City Metro	219,584	9,323	5.48	22
	Cacadu	129,937	5,338	3.35	22
	Chris Hani	199,122	11,875	4.40	22
	Joe Gqabi	85,342	3,740	6.58	22
	Nelson Mandela Bay Metro	351,128	14,890	3.40	22
	O R Tambo	307,836	7,468	4.53	22
**Free State**	Fezile Dabi	142,568	8,585	2.75	23.7
	Lejweleputswa	177,529	8,958	3.77	23.7
	Mangaung Metro	225,174	12,505	2.35	23.7
	Thabo Mofutsanyana	187,981	15,166	1.78	23.7
	Xhariep	34,386	2,371	1.81	23.7
**Gauteng**	City Of Johannesburg Metro	1,398,976	65,578	8.87	18.8
	City Of Tshwane Metro	909,299	35,540	8.46	18.8
	Ekurhuleni Metro	919,875	36,940	10.25	18.8
	Sedibeng	266,413	7,473	9.66	18.8
	West Rand	247,815	11,392	9.16	18.8
**KwaZulu-Natal**	Amajuba	124,953	6,769	6.60	30.1
	Ethekwini Metro	973,272	69,947	4.48	30.1
	Ilembe	166,987	10,221	3.95	30.1
	Sisonke	108,876	6,908	4.91	30.1
	Ugu	182,280	13,470	3.45	30.1
	Umgungundlovu	289,763	17,996	6.70	30.1
	Umkhanyakude	144,256	8,343	3.69	30.1
	Umzinyathi	126,826	7,088	4.66	30.1
	Uthukela	170,332	9,607	6.78	30.1
	Uthungulu	230,257	19,211	4.29	30.1
	Zululand	188,791	11,437	5.96	30.1
**Limpopo**	Capricorn	315,729	23,522	3.23	16.3
	Greater Sekhukhune	286,625	15,248	3.52	16.3
	Mopani	299,994	17,254	4.21	16.3
	Vhembe	350,588	15,561	3.44	16.3
	Waterberg	180,369	10,056	4.25	16.3
**Mpumalanga**	Ehlanzeni	406,003	26,559	9.47	23.6
	Gert Sibande	262,561	12,234	9.03	23.6
	Nkangala	368,211	16,326	7.05	23.6
**North West**	Bojanala	379,054	24,370	4.60	21.1
	Dr Kenneth Kaunda	195,631	14,381	4.30	21.1
	Dr Ruth Segomotsi Mompati	111,085	9,728	2.22	21.1
	Ngaka Modiri Molema	220,569	11,322	3.25	21.1
**Northern Cape**	Frances Baard	101,320	5,615	2.19	12.5
	John Taolo Gaetsewe	58,442	2,263	1.28	12.5
	Namakwa	34,418	1,273	2.99	12.5
	Pixley Ka Seme	50,012	1,627	2.09	12.5
	Siyanda	65,305	1,744	2.64	12.5
**Western Cape**	Cape Winelands	232,318	18,543	5.06	6.8
	Central Karoo	20,258	1,485	3.03	6.8
	City Of Cape Town Metro	1,136,697	80,790	4.45	6.8
	Eden	175,508	14,950	3.30	6.8
	Overberg	79,324	5,520	4.24	6.8
	West Coast	125,393	7,126	4.13	6.8

## 4. Discussion

### 4.1. Pap Smear Coverage

We found that Pap smear screening coverage is well below 50% in the HIV-infected population, indicating a significant gap in access to care for this population. Women infected with HIV are at increased risk of HPV infection and progression of cervical pre-cancer and cancer [1]; therefore, increased capacity for cervical cancer screening is urgently needed through introduction of interventions that increase uptake of screening, as well as those to improve availability and accessibility of services. As low coverage rates were more common in rural districts, interventions may need to include deploying mobile sites in hard to reach areas [16], even if at higher cost than facility-based screening. Many women in South Africa are aware of Pap smears, but may not understand the frequency of testing required or how to access screening. A study that examined cervical cancer awareness and practices among women sampled from a local clinic in Johannesburg, South Africa showed that although 71% of participants reported to be aware of Pap smear screening, only 37% and 29% of women had adequate Pap smear screening practice according to the national cervical cancer and HIV guidelines, respectively [17]. A study conducted at a tertiary institution showed 60% of respondents knew about HPV [18] and another urban based study showed that 59% of women had had a Pap smear [19]. If we explore awareness and knowledge of Pap smears in the community, one study conducted in Cape Town showed that over 50% of women had never had a Pap smear or had 1 more than 10 years ago [20]. Knowledge and awareness of Pap smears is more evident in urban areas and in women with a high level of education. In the rural areas and in local communities, more research needs to be conducted to assess their level of awareness and the results could be used to develop targeted interventions. Communication and awareness raising that involves the community, community health workers and by using appropriate media such as the radio, could result in improved coverage rates [16].

Coverage rates reported are based on individual public sector clinics or facility monthly reports. Our estimates are lower than those reported in the South African District Health Barometer 2013/14 [21] which may indicate under reporting of the counts of women aged 30 years and older presenting at the facilities or demonstrates how the facilities are under performing in screening the target population.

### 4.2. Pap Smear Quality

Only six districts performed at or above the national adequacy standard; thus, there is a need for further evaluations and interventions to improve adequacy rates. With low adequacy rates, a large number of Pap smears may have to be repeated or a large number of women may have been issued with false negative results thus reducing the benefits of screening for cervical cancer. Adequacy rates could be increased at the health facility level through additional staff training on accurate collection of cervical cells or the specificity of collection devices used in the public sector should be examined as they could result in inadequate sampling or inefficient recording of endocervical cells under the microscope. It has been previously demonstrated that by using a plastic Cervex brush (“broom”) not only results in increased adequacy rates, but it is also cost saving when compared to the wooden spatula [22].

### 4.3. High-grade Abnormalities

There are disparities in the burden of high-grade cytology results across South Africa. Although we are not able to directly address why this is, we speculate that the observed disparity may be attributed to the varying HPV and/or HIV prevalence rates across the districts. Furthermore, Pap smear laboratory reading and quality assurance may vary by district which may influence the variability of high-grade abnormalities. Other factors that influence this variability include HIV status, access to early treatment and age of screening. HPV testing in South Africa is not yet widespread and therefore data on the prevalence of HPV or on high-risk HPV types by district was not available for our analysis. Future research may look to map whether differences in the prevalence of high-risk HPV types are correlated with the geographic disparity in high-grade cytology results. Given the association between HIV and risk of progression for cervical disease, this disparity also may be attributed to the varying HIV prevalence rates for women across the districts.

Additionally, differential rates of access to cervical cancer prevention services likely play an important role. Districts with a high burden of high-grade cervical cytology abnormalities may require additional resource allocation, which may include ensuring that regions have colposcopy facilities as a previous study has demonstrated that the sensitivity of detecting HSIL increases if multiple biopsies are done [23]. Additional improvements that can be done also include increasing clinical providers trained in cervical colposcopy and both excisional and ablative treatments of cancer pre-cursors in high burden districts in order to reduce the burden [24]. By investing in early treatment of cancer pre-cursors, the incidence of cervical cancer can be greatly reduced [6].

### 4.4. Limitations

This study used facility-level counts of Pap smears as examined by the NHLS. Approximately 80% of the population access public healthcare and is not specific to gender or age therefore for this study, the population is unadjusted to eliminate introduction of uncertainty. As a result, Pap smear coverage reported may be an underestimate. Patient-level data was not available and therefore associations with patient-level characteristics such as age and HIV status could not be analysed. Further, the dataset did not allow determination of the total number of times one woman presented for Pap smear during the year. If the average woman included in the NHLS database received more than one Pap smear during the year, coverage rates presented here would be overestimated.

The coverage rate for HIV infected women in the NHLS data base is recorded on an individual's self-reporting of HIV status which might vary from the true HIV status therefore, the Pap smear coverage in HIV infected women reported in this report can deviate from the true coverage rate. It must be noted that the adequacy rates and high grade cytological abnormalities results documented have been used as proxies to measure the true quality and burden of cervical cancer.

## 5. Conclusion

Using choropleth maps, we are able to visually display three important components of a cervical cancer screening programme: (1) coverage of Pap screening services; (2) quality of Pap smears collected; and (3) burden of high-grade cytological abnormalities. Our study demonstrates there are observed differences in Pap smear coverage and adequacy and these results may be used to inform policy, programming and resource allocation. In South Africa, there are no district level choropleth maps for cervical cancer and this study successfully used local data to report and display results on a district level of granularity. By using routinely collected facility data, we are able to highlight districts that need clinical support and resource allocation in order to improve cervical cancer screening and treatment. From the choropleth maps displayed, we can see that urgent interventions are needed to improve coverage and quality of cervical screening, and observed regional disparities in cytology results should be addressed. We recommend that the study is repeated in yearly intervals to explore if reporting cervical cancer results on choropleth maps impacts the districts to improve on their screening program and to examine how the coverage is performing in HIV infected women following the revision of the screening guidelines which now recommend annual screening in HIV infected women.
